# Dynamic nonlinear control strategies for resilient heterogeneous vehicle platooning and handling Byzantine attacks in communication networks

**DOI:** 10.1016/j.heliyon.2024.e41574

**Published:** 2025-01-06

**Authors:** Ammar Alsinai, José Roberto Castilho Piqueira, Waqar Ul Hassan, Azmat Ullah Khan Niazi, Farida Safder

**Affiliations:** a Department of mathematics, CV Raman Global University, Odisha, India; b Systems engineering, Escola Politécnica da USP, São Paulo, Brazil; c Department of Mathematics and Statistics, The University of Lahore, Sargodha 40100, Pakistan

**Keywords:** Nonlinear control method, Inter-linkage between cars (leader-follower framework), Byzantine attacks, Heterogeneous dynamic model, Lyapunov function approach, Constant time headway spacing policy

## Abstract

This paper addresses the challenges of maintaining stability in heterogeneous vehicle platooning under the influence of communication disruptions caused by Byzantine attacks within a leader-follower framework. To enhance resilience, we propose a nonlinear control strategy tailored for a third-order heterogeneous dynamic model, integrating leader-follower interconnections with adaptable control gains. Utilizing a constant time headway spacing policy with gap adjustments, we derive control gains that secure internal platoon stability. A Lyapunov-based stability approach is employed to mitigate the destabilizing effects of Byzantine attacks, ensuring robust performance even in adverse conditions. Numerical simulations validate the controller's effectiveness, showing rapid convergence to stable platoon behavior within 10 seconds and maintaining a tracking error below 10 percent, despite simulated attacks. These results demonstrate the practical feasibility of the proposed controller in ensuring stability and resilience in real-world platooning scenarios where communication integrity is compromised.

The graphical representation of abstract of this paper is shown in Fig. 1.

**Figure 1:**
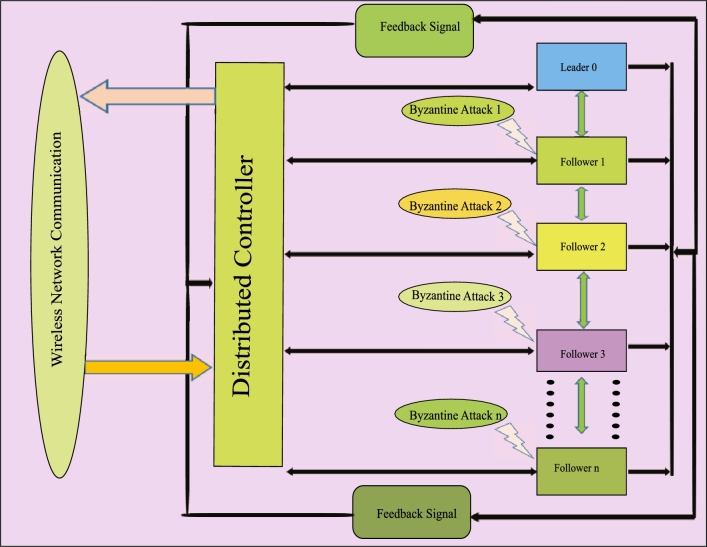
Graphical representation of abstract.

## 1 Introduction

To make a secure functioning and proper working vehicle platooning by using a control system plays a vital and very effective role especially when dealing with the disruptive presence of hostile vehicles within the network [1]. These vehicles represent a significant risk due to their disregard for communication norms and their transmission of false information [2], [3]. This fraudulent behavior is commonly known as “Byzantine assaults,” which presents a serious risk to platoon coordination and may cause accidents that threaten infrastructure and human life [4]. To stop harmful actions and other challenges, it's critical to develop novel control strategies, algorithms, and detection techniques [5] which are helpful for networking of CvSs. The efficiency of these controls determines the overall security and safety of platoon systems. Conventional control methods become useless because of the inherent complexity and decentralized nature of Intelligent Connected Vehicles (ICVs). However, [6], [7], a conceptual change in support of distributed control techniques is necessary. It is essential to develop a resilient distributed platoon control system [8]. A robust and adaptable framework is essential to rapidly detect and eliminate cyberattack risks [9], [10]. By applying robust algorithms and solid-based techniques for abnormal acceptance and behavior analysis, it may be possible to successfully decrease challenges associated with hostile living things within the network [11]. Furthermore, it is also essential that the distributed control system design such type of prioritizes reliability and fault tolerance to ensure that platoon operations persist continuously even in the event of malfunctioning nodes or communication links fail [12], [13]. Consensus-based decision-making can enhance platoon cooperation and mitigate the impact of hostile acts [14]. By doing continuous research, development efforts, and particularly our practical implementations are essential to maintain a competitive edge against developing cyber-security threats [15]. In order to improve security and transparency inside ICV networks, it is crucial to consistently improve controls while testing novel technologies and experiments, including the decentralized ledger [16], [17]. The development of a strong distributed platoon control system requires continuous creativity and collaboration across disciplines [18]. Through the implementation of complex leadership methods, vigilant observation, and versatile reaction mechanisms, we can protect platoon systems against the constant risk of cyberattacks, leading to a safer and better traveling environment [19]. Adaptive Federated Learning (AFL) has been used to wireless networks in several areas of research [20], [21]. In order to maximize throughput presented an original approach that combined deep Q-learning with AFL, stressing the constant need for highly reliable communication with minimal delay. Meanwhile, researchers presented a novel Federated Learning (FL) system with an autonomous update and also intended to reach low latency and high efficiency in splitting resources across various user groups [22], [23]. Similarly, Liu et al. devised an AFL arbitration framework integrating bidirectional long short-term memory (LSTM) and an attention mechanism to enhance model accuracy and reduce communication rounds. Additionally, Wang et al. introduced a centralized fusion algorithm within their AFL scheme, aimed at enhancing training efficiency [24], [25]. Meanwhile, Lee et al. proposed an adaptive scheduling strategy leveraging AFL within wireless distributed learning networks [26]. However, none of these studies accounted for the unique characteristics of vehicles, such as mobility, data size, and computational capabilities, when selecting participants for AFL in vehicular networks.

On the other hand, several research efforts have addressed Byzantine attacks in wireless networks [27]. For example, Vedant et al. proposed a method integrating practical Byzantine fault tolerance to enhance privacy and security in energy exchanges between electric vehicles and the grid. Ma et al. introduced a credibility-based mechanism and an efficient protocol to protect privacy during gradient aggregation, specifically focusing on Byzantine attacks within Non-IID datasets collected from heterogeneous ships. Meanwhile, Sheikh et al. concentrated on securing the energy exchange process between electric vehicles and the power grid within a blockchain consensus system, leveraging Byzantine mechanisms to bolster security [28], [29]. Additionally, Wang et al. presented a decentralized system for securely collecting traffic data and preserving privacy using blockchain technology, along with fog/edge computing infrastructure capabilities [30]. Huang et al. proposed a method to integrate vehicle spacing data for estimating traffic density, with the aim of resisting Byzantine attacks [31]. Similarly, Chen et al. introduced a novel Byzantine fault-tolerant FL scheme wherein each vehicle utilized a publicly-verifiable secret-sharing mechanism to safeguard model confidentiality [32]. Meanwhile, Wang developed a decentralized system for securely collecting traffic data and protecting privacy on the blockchain, while employing DL techniques to mitigate Byzantine and provided a networking approach resilient to Byzantine faults, alongside resource allocation strategies for fog computation-enabled internet of things [33]. Lastly, Wang et al. proposed integrating vehicle spacing data to estimate average spacing and devised various estimation methods to counter different types of Byzantine attacks [34]. However, none of these studies addressed the integration of AFL into vehicular networks. Cyber-attacks targeting communication channels pose significant risks to Information is secure, reliable, and accessible [35]. In response to danger, numerous robust control strategies have been developed for Intelligent Connected Vehicles (ICVs) to mitigate the diverse impacts of cyber threats, including denial-of-service (DoS), deception, and eavesdropping attacks [36]. For example, researchers have examined the effects of DoS attacks on ICVs, which often lead to time delays [37]. They have utilized a combination of adaptive estimation and sliding mode control techniques to identify and assess the severity of such attacks. Additionally, resilient cooperative adaptive cruise control systems have been designed to address DoS attacks characterized by packet dropouts, establishing the resilience threshold against consecutive packet dropouts. Recent studies have also investigated deception attacks targeting assaults on data integrity, including replay and fake data instillation (FDI) [38]. To counter replay attacks, a dynamic tracking controller integrates output feedback control with a robust reset control mechanism to mitigate significant random communication delays caused by such attacks. Regarding FDI attacks, two notable detection mechanisms have been proposed: one leveraging a cloud-based sandboxing technique to evaluate and distinguish between hostile assaults in ICV circumstances and the other utilizing an observer based on partial differential equations to identify FDI assaults and pinpoint their injection sites within the ICV platoon. Additionally, advancements have been made in controls that protect security for ICVs, particularly in relation to the security of information.

In recent years, significant advancements have been made in optimizing communication and control strategies for dynamic systems such as vehicle platooning [39]. For instance, the research on self-adaptive multi-strategy optimization in wireless sensor networks emphasizes the importance of avoiding local optima, a challenge that is directly relevant to ensuring robust communication in vehicle platooning systems under disruptive conditions like Byzantine attacks [40]. Additionally, work on task offloading and resource allocation in B5G/6G edge networks has explored the optimization of communication networks in mobile environments [41], which shares similarities with the challenges faced by vehicle platooning systems in managing resources and maintaining communication reliability amidst high mobility and potential communication failures. In the context of real-time adaptation [42], a study on taxi demand prediction using deep spatial-temporal networks highlights the need for dynamic adjustments, a principle that applies to vehicle platooning systems where vehicle dynamics must be continuously monitored and controlled [42]. Finally, research on secure feedback systems based on blockchain for the Internet of Vehicles demonstrates innovative solutions for ensuring communication security, an essential component in maintaining stability and robustness in platooning systems vulnerable to attacks [43]. These studies, though focused on different areas, collectively underline the need for advanced control and communication strategies that can adapt to dynamic conditions, a need that is central to the current work on enhancing the stability and resilience of heterogeneous vehicle platooning systems.

Motivated by our previous discussion, we outlined contributions in a way that highlights the critical need for addressing vulnerabilities in vehicle platooning systems.

In recent years, vehicle platooning has gained significant attention as a promising solution for improving road safety, fuel efficiency, and traffic flow. However, one of the major challenges in platooning systems is the vulnerability to communication disruptions, particularly those caused by Byzantine attacks, which can undermine the integrity and stability of the platoon. While previous studies, such as [44] and [45] have explored control strategies for heterogeneous vehicle platoons, none have fully addressed the issue of Byzantine attacks within such systems. This research fills this gap by introducing a novel nonlinear control approach that integrates car-following dynamics with a third-order heterogeneous vehicle model, ensuring platoon stability even in the presence of malicious data injection. The proposed technique enhances the resilience of the platooning system, offering robust performance despite communication disruptions and malicious attacks.

The structure of the remaining content is as follows:

1. In the section 1, we present and investigate the implications of Byzantine assaults on the controller from multiple perspectives.
2. In the section 2 graph theory explained for the communication of agents.
3. In the section 3, we present the mechanism of information flow for n-vehicle platooning.
4. In the section 4, we represent the problem formation model and feedback linearization method.
5. In section 5, we designed a distributed controller under the effect of Byzantine attack and also provided some lemmas, a dynamic tracking system, and also provided theorems for the proof of our theoretical model.
6. In section 6, we provided numerical simulations for the proof of above main results for five heterogeneous vehicle platooning, at the end of figures, table also provided with the comparison existing research, discussion of numerical simulations, discussion on computational complexity and feasibility and also practical implications for real world implementation provided.
7. In section 7, we made some conclusions.

## 2 Preliminaries

### 2.1 Communication typologies

In the realm of Intelligent Connected Vehicles (ICVs), information exchange is depicted through a directed graph Γ=(V,E,A), where $V=\{v_{1},v_{2},...,v_{M}\}$ represents the vertex set with *M* vehicles. The set of edges $E(\overset{˜}{y})$ at time *t* comprises pairs $\left(v_{i},v_{j}\right)$, where $v_{i},v_{j}\in V$ and i≠j, signifying communication links. The adjacency matrix $A(\overset{˜}{y})$, of size M×M, denotes the strength of connectivity between vehicles. An entry $a_{ij}(\overset{˜}{y})>\alpha$ indicates that vehicle $v_{j}$ can transmit data to vehicle $v_{i}$ at time $\overset{˜}{y}$, where 0<α<1. The in-degree $\delta_{i}$ of vehicle $v_{i}$ is computed as the sum of incoming edges from other vehicles. Represented as D(t), the in-degree matrix is diagonal with entries $\delta_{1},\delta_{2},...,\delta_{M}$. The Laplacian matrix $L(\overset{˜}{y})$ is derived as $L(\overset{˜}{y})=D(\overset{˜}{y})-A(\overset{˜}{y})$, characterizing the network topology. The neighbors of vehicle $v_{i}$, denoted by $N_{i}$, encompass vehicles $v_{j}$ such that $\left(v_{i},v_{j}\right)$ is an edge at time $\overset{˜}{y}$. In this configuration, only a subset of Information can be sent to the following cars from a leader vehicle $v_{0}$. The pinning matrix G is diagonalized with elements $g_{10},g_{20},...,g_{M0}$, representing pinning gains. Each follower vehicle $v_{i}$ gathers data solely from the previous cars $v_{i-1}$ and $v_{i-2}$, where *i* ranges from 2 to *M*.

Definition 2.1 Given a directed graph Γ=(V,E,A) and a positive integer *F*, if for each normal vehicle $v_{i}\in V_{N}$, the number of Byzantine vehicles in its neighborhood, denoted by $N_{i}\cap V_{A}$, is no more than *F*, then the ICV is said to be subject to *F*-local Byzantine attacks.

## 3 Mechanism of information flow

In a convoy, there are *n* heterogeneous vehicles, denoted as $V_{0},V_{1},\ldots ,V_{n}$, where $V_{i}$ (for i∈{1,2,…,n}) signifies the *i*-th vehicle, while $V_{0}$ is identified as the leading vehicle. Let $L_{i}$ denote the length of vehicle $V_{i}$. The position, speed, and rate of change of speed of $V_{i}$ at time $\overset{˜}{y}$ are respectively represented by $x_{i}(\overset{˜}{y})$, $s_{i}(\overset{˜}{y})$, and ${\overset{˙}{s}}_{i}(\overset{˜}{y})$.

## 4 Problem formation model

The dynamics of each vehicle *i* (where i∈n) explained by the following system,

(1) $$\left\{ \begin{array}{c} {\overset{˙}{p}}_{i}(\overset{˜}{y})=s_{i}(\overset{˜}{y})\\ {\overset{˙}{s}}_{i}(\overset{˜}{y})=\frac{1}{m_{i}}\left(\xi_{u,i}\frac{u_{i}(\overset{˜}{y})}{r_{i}}-L_{i}s_{i}{\left(\overset{˜}{y}\right)}^{2}-m_{i}a_{g}h\right)\\ \gamma_{i}{\overset{˙}{U}}_{i}(\overset{˜}{y})+U_{i}(\overset{˜}{y})=U_{i,\text{des}}(\overset{˜}{y}). \end{array}\right.$$

Where $m_{i}$ represents the vehicle mass, $\xi_{U,i}$ is the mechanical efficiency, $U_{i}(\overset{˜}{y})$ and $U_{i,\text{des}}(\overset{˜}{y})$ denote the real and intended torque for braking and driving respectively, $L_{i}$ is the coefficient of lumped aerodynamic drag, $r_{i}$ stands for the tire radius, $a_{g}$ represents the gravitational acceleration, h is the rolling resistance coefficient, and $\gamma_{i}$ indicates the inertia delay of the longitudinal dynamics.

### 4.1 Feedback linearization method

By using this method [46],

(2) $$U_{i,des}(\overset{˜}{y})=\frac{r_{i}}{\xi_{U,i}}[(L_{i}s_{i}(\overset{˜}{y})+m_{i}g_{a}h)(s_{i}+2\overset{˙}{s_{i}}\gamma_{i})+\zeta_{i}(\overset{˜}{y})m_{i}].$$

By using the Eq (2) on Eq (1) can be obtained,

(3) $$\xi_{i}\overset{˙}{a_{i}}+a_{i}(\overset{˜}{y})=\zeta_{i}(\overset{˜}{y}).$$

Where $\zeta_{i}(\overset{˜}{y})$ is the control input, and $\xi_{i}$ shows the heterogeneous type of vehicles.

We can obtain leader and follower vehicle dynamics from equation (1) as

(4) $${\overset{˙}{p}}_{i}(\overset{˜}{y})=s_{i}(\overset{˜}{y}).$$

(5) $${\overset{˙}{s}}_{i}(\overset{˜}{y})=a_{i}(\overset{˜}{y}).$$

(6) $$\xi_{i}{\overset{˙}{a}}_{i}(\overset{˜}{y})+a_{i}(\overset{˜}{y})=\zeta_{i}(\overset{˜}{y})+b_{i}(\overset{˜}{y}).$$

Where $b_{i}(\overset{˜}{y})$ is Byzantine attack. Similarly, we can write dynamics for leader vehicles,

(7) $${\overset{˙}{p}}_{0}(\overset{˜}{y})=s_{0}(\overset{˜}{y}).$$

(8) $${\overset{˙}{s}}_{0}(\overset{˜}{y})=a_{0}(\overset{˜}{y}).$$

(9) $$\xi_{0}{\overset{˙}{a}}_{0}(\overset{˜}{y})+a_{0}(\overset{˜}{y})=\zeta_{0}y_{0}(\overset{˜}{y}).$$

In this analysis, the constant time headway spacing policy is utilized to define the optimal distance between vehicles on the roadway, ensuring a consistent temporal interval between successive vehicles for heightened traffic safety and efficiency. The preferred gap between adjacent vehicles denoted as S, facilitates safe braking and reaction times. Where $S_{i,i-1}(\overset{˜}{y})$ represents the dynamic spacing between vehicles at time $\overset{˜}{y}$, adherence to this distance empowers drivers to regulate their speed and uphold safe following distances, thereby improving traffic management and mitigating congestion.

$$S_{i,i-1}=S+Cs_{i}(\overset{˜}{y}).$$

Where C>0 is constant time headway and S>0 is a constant distance. The spacing error between the vehicles can be expressed as $e_{i}(\overset{˜}{y)}=p_{i-1}(\overset{˜}{y})-p_{i}(\overset{˜}{y})-L_{v}-(S+Cs_{i}(\overset{˜}{y}))$.

## 5 Designing of controller

**Figure 2:**
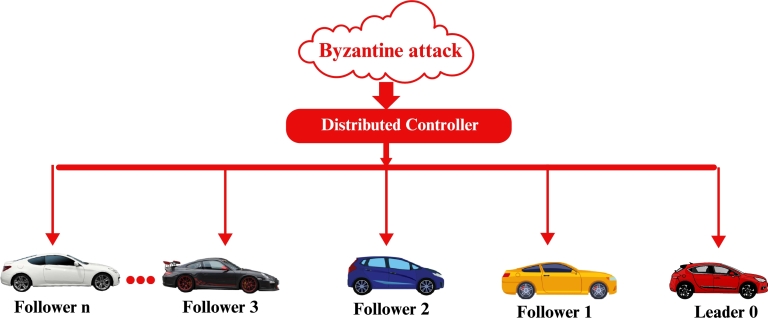
Network communication topology of nonlinear heterogeneous vehicle platooning under the Byzantine attack.

We design a heterogeneous i=0,1,2,3,...,n car-following dynamics to ensure vehicles follow traffic flow principles which are shown in Fig. 2. The control inputs are designed as $\zeta_{i}(\overset{˜}{y})=\zeta_{i}^{\text{P}}(\overset{˜}{y})+\zeta_{i}^{\text{F}}(\overset{˜}{y})+\zeta_{i}^{\text{L}}(\overset{˜}{y})+b_{i}(\overset{˜}{y})$. Here, $\zeta_{i}^{\text{P}}(\overset{˜}{y})$ is the control input based on data from vehicles in front, helping the controlled vehicle respond to changes in their speed and position. $\zeta_{i}^{\text{F}}(\overset{˜}{y})$ comes from the information of vehicles behind, ensuring safe distances and smooth flow. Furthermore, $b_{i}(\overset{˜}{y})$ accounts for potential Byzantine attacks, mathematically defined as $b_{i}(\overset{˜}{y})=\sum_{k\in B}b_{k}^{\text{P, F}}(\overset{˜}{y})$, indicating the impact of malicious vehicles injecting false data. Combining these elements, $\zeta_{i}(\overset{˜}{y})$ ensures a robust and comprehensive control strategy. This approach maintains appropriate distances and speeds relative to neighboring vehicles while being resilient to malicious disruptions.

We define the desired spacing distance for i > j,

(10) $$S_{ij}(\overset{˜}{y})=(i-j)S+C\sum_{F=j+1}^{i}s_{F}(\overset{˜}{y}).$$

Similarly, the desired spacing distance for i < j,

(11) $$S_{ij}(\overset{˜}{y})=-(i-j)S-C\sum_{F=i+1}^{j}s_{F}(\overset{˜}{y}).$$

When the optimum path between the i-th car and the leader vehicle,

(12) $$S_{i0}(\overset{˜}{y})=\sum_{F=1}^{i}(Cs_{F}(\overset{˜}{y})+L_{f}+S).$$

By using the Eq (3), Eq (4), Eq (5), Eq (6), Eq (7), Eq (8), Eq (10), Eq (11) and Eq (12), we design a controller that enhances traffic flow efficiency and safety, reducing the risk of traffic jams and accidents. By considering Byzantine attacks, the system is better equipped to handle disruptions, resulting in a more stable and efficient,

(13) $$\zeta_{i}(\overset{˜}{y})=\zeta_{i}^{\text{P}}(\overset{˜}{y})+\zeta_{i}^{\text{F}}(\overset{˜}{y})+\zeta_{i}^{\text{L}}(\overset{˜}{y})+b_{i}(\overset{˜}{y})=-\sum_{j=1}^{n}b_{ij}\left[K_{i,u}(s_{i}(\overset{˜}{y})-O_{v_{i}}(\Delta p_{ij}(\overset{˜}{y})))+K_{i,s}(s_{i}(\overset{˜}{y})-s_{j}(\overset{˜}{y})\right)\;+K_{i,p}(p_{i}(\overset{˜}{y})-p_{j}(\overset{˜}{y}))-s_{0}(\overset{˜}{y})+S_{ij}(t))]\;-R(i,i)\left[K_{0,s}(s_{i}(\overset{˜}{y})-s_{0}(\overset{˜}{y}\right)\;+K_{0,p}(p_{i}(\overset{˜}{y})-p_{0}(\overset{˜}{y})-s_{0}(\overset{˜}{y})+S_{i0}(t))]+\sum_{k\in B}b_{k}^{\text{P, F}}(\overset{˜}{y}).$$

Where $K_{i,u}\in R^{+}$, $K_{i,p}\in R^{+}$, $K_{i,s}\in R^{+}$, $K_{0,p}\in R^{+}$, and $K_{0,s}\in R^{+}$ are control gains. Now we define the optimal velocity for the follower vehicles such that $O_{i}(\Delta_{ij}(\overset{˜}{y}))=O_{1}+O_{2}tanh(P_{1}\Delta p_{ij}(\overset{˜}{y})-P_{1})$ Where $O_{1}\in R$ and $O_{2}\in R$, representing real-valued constants in the optimal velocity function for follower vehicles and it also based on desired system performance metrics, such as stability and convergence speed. Where $P_{1}$ and $P_{2}$ represent scaling factors. Now we define the state equation for the third-order model of the *i*th vehicle is and using the Eq (13),

(14) $${\overset{˙}{z}}_{i}(\overset{˜}{y})=M_{i}z_{i}(\overset{˜}{y})+N_{i}[(\zeta_{i}(\overset{˜}{y})=\zeta_{i}^{\text{P}}(\overset{˜}{y})+\zeta_{i}^{\text{F}}(\overset{˜}{y})+\zeta_{i}^{\text{L}}(\overset{˜}{y})+b_{i}(\overset{˜}{y}))].$$

In this equation, ${\overset{˙}{z}}_{i}(\overset{˜}{y})$ represents the rate of change of the state vector $z_{i}(\overset{˜}{y})$ of the *i*th vehicle. The matrix $M_{i}$ applies to the state vector to describe the system's inherent dynamics, while the matrix $N_{i}$ and the control input $\zeta_{i}(\overset{˜}{y})$ influence the vehicle's state. Where $z_{i}(\overset{˜}{y})=\left[\begin{array}{c} p_{i}(\overset{˜}{y})\\ s_{i}(\overset{˜}{y})\\ a_{i}(\overset{˜}{y}) \end{array}\right],\;M_{i}=\left[\begin{array}{ccc} 0 & 1 & 0\\ 0 & 0 & 1\\ 0 & 0 & -\frac{1}{\xi_{i}} \end{array}\right],\;N_{i}=\left[\begin{array}{c} 0\\ 0\\ \frac{1}{\xi_{i}} \end{array}\right]$. Define the tracking errors for position, velocity and acceleration such that ${\overset{˜}{p}}_{i}(\overset{˜}{y})=p_{i}(\overset{˜}{y})-p_{i}^{e}(\overset{˜}{y}),{\overset{˜}{s}}_{i}(\overset{˜}{y})=s_{i}(\overset{˜}{y})-s_{i}^{e}(\overset{˜}{y})\;\;and\;\;{\overset{˜}{a}}_{i}(\overset{˜}{y})=a_{i}(\overset{˜}{y})-a_{i}^{e}(\overset{˜}{y})$. Now we take the state $\overset{˜}{z}(\overset{˜}{y})={\left[{\overset{˜}{p}}_{i}(\overset{˜}{y}),{\overset{˜}{s}}_{i}(\overset{˜}{y}),{\overset{˜}{a}}_{i}(\overset{˜}{y})\right]}^{T}$ We assume and take leader vehicle i=0 with constant velocity.

### 5.1 Dynamic tracking system

Let $\overset{˜}{z}(\overset{˜}{y})=[{\overset{˜}{z}}_{1}^{T}(\overset{˜}{y}),...,{\overset{˜}{z}}_{n}^{T}$]. Because there is only one leader and *n* follower vehicles and solving the Eq (14). So, we can design dynamics in matrix form such that,

(15) $$\overset{˙}{\overset{˜}{z}}(\overset{˜}{y})=E_{0}\overset{˜}{z}(\overset{˜}{y})+\sum_{r=1}^{n}E_{r}\overset{˜}{z}(\overset{˜}{y}).$$

Where we take the matrix such that,

$$E_{0}=\left(\begin{array}{ccc} 0_{n} & E_{n} & J\\ 0_{n} & 0_{n} & 0_{n}\\ -\rho X_{1} & -\rho X_{2} & -\rho \end{array}\right)\;and\;\;E_{r}=\left(\begin{array}{ccc} 0_{n} & E_{n} & 0_{n}\\ 0_{n} & 0_{n} & 0_{n}\\ -\rho X_{6} & -\rho X_{7} & -0_{n} \end{array}\right).$$

Where,

$$\left\{ \begin{array}{c} \rho =\mathrm{diag}[\beta_{1},\beta_{2},\ldots ,\beta_{n}],\\ X_{1}=\mathrm{diag}[K_{i,u}]X_{3}+\mathrm{diag}[K_{i,p}]\mathrm{diag}[D]+K_{0,p}R,\\ X_{2}=\mathrm{diag}[D]\mathrm{diag}[K_{i,v}+K_{i,u}]+K_{0,v}R+C\mathrm{diag}[K_{i,u}X_{4}]+C\mathrm{diag}[K_{i,p}X_{5}],\\ X_{3_{ij}}=b_{ij}\psi_{ij},\\ X_{4_{ij}}=\sum_{k=1}^{n}b_{ik}\psi_{ik}-\sum_{k=1}^{j-1}b_{ik}\psi_{ik},\\ X_{5_{ij}}=\sum_{k=1}^{n}b_{ik}-\sum_{k=1}^{j-1}b_{ik},\\ X_{6}=\left\{ \begin{array}{cc} X_{3}K_{i,u}+b_{ij}K_{i,p}, & j\neq i\\ 0, & j\neq i\\ 0, & j=i \end{array}\right.,\\ X_{7}=\left\{ \begin{array}{cc} CK_{i,u}X_{4}+CK_{i,p}X_{5}, & j\neq i\\ 0, & j\neq i\\ 0, & j=1 \end{array}\right.. \end{array}\right..$$

Lemma 5.1 *In the domain of matrix theory, the determinant*det(M)*of a square matrix theory M which serves as a fundamental of its intrinsic behavior, when*det(M)*is expressed as the product of n distinct functions*$F_{i}(\mu )$*, the matrix*S*.*

$$\Sigma_{i=1}^{n}F_{i}(\mu )=\Sigma_{i=1}^{n}(\mu^{3}+c_{i}\mu^{2}+d_{i}\mu +e_{i}),\;i=1,2,\ldots ,n,$$

*where each*$F_{i}(\mu )$*stands for a cubic polynomial in μ, featuring coefficients*$c_{i}$*,*$d_{i}$*, and*$e_{i}$*for*i=1,2,…,n*.*

Lemma 5.2 *To ascertain the Hurwitz stability of a complex polynomial*F(μ)*, it is essential to assess its real*a(ν)*and imaginary*b(ν)*components, where*$H^{2}=-1$*. A crucial condition for Hurwitz stability is that*$c(0)d^{\prime }(0)-c^{\prime }(0)d(0)$*must be greater than zero. The polynomial pair*(C(ν),b(ν))*must exhibit interlacing behavior, meaning that*c(ν)*and*d(ν)*should alternatively cross the zero axis as ν ranges from negative infinity to positive infinity. This characteristic indicates the stable behavior of the polynomial across the entire spectrum.*

Now apply Newton-Leibniz formula on Eq (15), and we can solve it as,

(16) $$\overset{˙}{\overset{˜}{z}}(\overset{˜}{y})=E_{0}\overset{˜}{z}(\overset{˜}{y})+\sum_{r=1}^{n}E_{r}\overset{˜}{z}(\overset{˜}{y}).$$

Integrate both sides of Eq (16) with respect to $\overset{˜}{y}$ from ${\overset{˜}{y}}_{0}$ to $\overset{˜}{y}$,

(17) $$\int_{{\overset{˜}{y}}_{0}}^{\overset{˜}{y}}\overset{˙}{\overset{˜}{z}}(\overset{˜}{y})\;d\overset{˜}{y}=\int_{{\overset{˜}{y}}_{0}}^{\overset{˜}{y}}\left(E_{0}\overset{˜}{z}(\overset{˜}{y})+\sum_{r=1}^{n}E_{r}\overset{˜}{z}(\overset{˜}{y})\right)\;d\overset{˜}{y}.$$

Using the Newton-Leibniz formula,

(18) $$\overset{˜}{z}(\overset{˜}{y})-\overset{˜}{z}({\overset{˜}{y}}_{0}).$$

The right-hand side of Eq (17) becomes and we get,

(19) $$=E_{0}\int_{{\overset{˜}{y}}_{0}}^{\overset{˜}{y}}\overset{˜}{z}(\overset{˜}{y})\;d\overset{˜}{y}+\sum_{r=1}^{n}E_{r}\int_{{\overset{˜}{y}}_{0}}^{\overset{˜}{y}}\overset{˜}{z}(\overset{˜}{y})\;d\overset{˜}{y}.$$

Combining the results from Eq (18) and Eq (19) both sides, and we get,

(20) $$\overset{˜}{z}(\overset{˜}{y})-\overset{˜}{z}({\overset{˜}{y}}_{0})=E_{0}\int_{{\overset{˜}{y}}_{0}}^{\overset{˜}{y}}\overset{˜}{z}(\overset{˜}{y})\;d\overset{˜}{y}+\sum_{r=1}^{n}E_{r}\int_{{\overset{˜}{y}}_{0}}^{\overset{˜}{y}}\overset{˜}{z}(\overset{˜}{y})\;d\overset{˜}{y}.$$

By rearrange the Eq (20) to solve for $\overset{˜}{z}(\overset{˜}{y})$,

(21) $$\overset{˜}{z}(\overset{˜}{y})=\overset{˜}{z}({\overset{˜}{y}}_{0})+E_{0}\int_{{\overset{˜}{y}}_{0}}^{\overset{˜}{y}}\overset{˜}{z}(\overset{˜}{y})\;d\overset{˜}{y}+\sum_{r=1}^{n}E_{r}\int_{{\overset{˜}{y}}_{0}}^{\overset{˜}{y}}\overset{˜}{z}(\overset{˜}{y})\;d\overset{˜}{y}.$$

Where $S=E_{0}+\sum_{r=1}^{n}E_{q}$ and $Q=E_{r}E_{0}$,

$$S=\left(\begin{array}{ccc} 0_{n} & I_{n} & J\\ 0_{n} & 0_{n} & I_{n}\\ -\rho K_{P} & -\rho K_{S} & -\rho \end{array}\right)\;and\;Q=\left(\begin{array}{ccc} J\rho X_{6} & \rho X_{7} & 0_{n}\\ \rho X_{6} & \rho X_{7} & 0_{n}\\ -\rho^{2}X_{6} & -\rho^{2}X_{7} & 0_{n} \end{array}\right).$$

Theorem 5.3 *A heterogeneous platoon consisting of vehicles potentially affected by Byzantine attacks can maintain stability if specific inequalities are satisfied for each vehicle i. These conditions must account for the malicious behaviors introduced by Byzantine attack to ensure proper coordination within the platoon.*

(22) $$\left\{ \begin{array}{c} \chi_{i}\sin\left(\frac{\psi +\pi /2}{3}\right)<-\frac{CI_{n}(\alpha_{i})+I_{n}(\gamma_{i})-\sqrt{\delta_{i}}}{2}+b_{i}<\chi_{i}\sin\left(\frac{2\pi +\psi +\pi /2}{3}\right)\\ -\frac{CI_{n}(\alpha_{i})+I_{n}(\gamma_{i})+\sqrt{\delta_{i}}}{2}+b_{i}<\chi_{i}\sin\left(\frac{4\pi +\psi +\pi /2}{3}\right). \end{array}\right.$$

(23) $$\left\{ \begin{array}{c} {\left((CI_{n}(\alpha_{i}))+I_{n}(\gamma_{i})\right)}^{2}+4Q(\alpha_{i})>b_{i}^{2}\\ I_{n}^{2}(\alpha_{i})\leq \frac{4}{27\xi_{\sigma ,i}}{\left(CQ(\alpha_{i})+Q(\gamma_{i})\right)}^{3}+b_{i}\\ CQ(\alpha_{i})+Q(\gamma_{i})>0\\ CQ^{2}(\alpha_{i})+Q(\alpha_{i})\cdot Q(\gamma_{i})+CI_{n}^{2}(\alpha_{i})+I_{n}(\alpha_{i})\cdot I_{n}(\gamma_{i})>b_{i}. \end{array}\right.$$

*Where,*

$$\psi =\arcsin\left(\frac{9\sqrt{\xi_{\sigma ,i}I_{n}(\alpha_{i})}}{2(CQ(\alpha_{i})+Q(\gamma_{i}))\sqrt{3(CQ(\alpha_{i})+Q(\gamma_{i}))}}\right),$$

$$\chi_{i}=\frac{2\sqrt{3(CQ(\alpha_{i})+Q(\gamma_{i}))}}{3\sqrt{\xi_{\sigma ,i}}},$$

$$\delta_{i}={\left(CI_{n}(\alpha_{i})+I_{n}(\gamma_{i})\right)}^{2}+4Q(\gamma_{i}).$$

*Where* $b_{i}$ *represents the Byzantine effect on vehicle i.*

Proof We start by considering the determinant of the matrix S,

$$S=\left|\begin{array}{ccc} \mu I_{n} & -I_{n} & -J+b_{i}\\ 0_{n} & \mu I_{n} & -I_{n}+b_{i}\\ \rho K_{P} & \rho K_{V} & \mu I_{n}+\rho +b_{i}. \end{array}\right|$$

Taking the determinant of S, and we get,

$$\det(S)=\left|\begin{array}{ccc} \mu I_{n} & -I_{n} & -J+b_{i}\\ 0_{n} & \mu I_{n} & -I_{n}+b_{i}\\ \rho K_{P} & \rho K_{V} & \mu I_{n}+\rho +b_{i} \end{array}\right|$$

Next, by using Lemma 5.1, Lemma 5.2, and we have,

$$\sum_{i=1}^{n}\left(\mu^{3}+\frac{1}{\xi_{i}}\mu^{2}+\left(C\alpha_{i}+\gamma_{i}\right)\frac{1}{\xi_{i}}\mu +\frac{\alpha_{i}}{\xi_{i}}\right).$$

We take,

$$F_{i}(H\nu )=-H\nu^{3}-\frac{1}{\xi_{i}}\nu^{2}+\frac{1}{\xi_{i}}(Q(\alpha_{i})+HI_{n}(\alpha_{i}))+\frac{C\left(Q(\alpha_{i})+HI_{n}(\alpha_{i})\right)+\left(Q(\gamma_{i})+HI_{n}(\gamma_{i})\right)+b_{i}}{\xi_{i}}H\nu .$$

By using Lemma 5.2, we take the real and imaginary parts of $F_{i}(H\nu )$, which we denote by $c_{i}(\nu )$ and $d_{i}(\nu )$,

$$\left\{ \begin{array}{c} c_{i}(\nu )=\frac{1}{\xi_{i}}\left(-\nu^{2}-(CI_{n}(\alpha_{i})+I_{n}(\gamma_{i}))\nu +Q(\alpha_{i})+b_{i}\right)\\ d_{i}(\nu )=\nu^{3}+\frac{HQ(\alpha_{i})+Q(\gamma_{i})+b_{i}}{\xi_{i}}. \end{array}\right.$$

Since we have $\delta_{i}>0$, then $c_{i}(\nu )=0$ and $d_{i}(\nu )=0$. Both Lemma 5.1, Lemma 5.2 assure the stability of our function $F_{i}(\mu )$. The modified inequalities and conditions stated are satisfied, accounting for the Byzantine effect, which completes the proof. □

Theorem 5.4 *If* S *is a Hurwitz matrix and there exists a constant* ${\overset{˜}{y}}^{⁎}>0$ *such that* $0<\overset{˜}{y}<{\overset{˜}{y}}^{⁎}$ *for all* r=1,…,n *, then the vehicle platoon described by Eq* (22) *and Eq* (23) *is said to be uniformly stable. This means that the platoon's state errors converge to zero as time approaches infinity, i.e.,*

$$\left\{ \begin{array}{c} \lim_{\overset{˜}{y}\to \infty }\|p(\overset{˜}{y})\|=0,\\ \lim_{\overset{˜}{y}\to \infty }\|s(\overset{˜}{y})\|=0,\\ \lim_{\overset{˜}{y}\to \infty }\|a(\overset{˜}{y})\|=0. \end{array}\right.$$

Proof Taking the Lyapunov candidate function,

$$L(z(\overset{˜}{y}))=z{\left(\overset{˜}{y}\right)}^{T}Pz(\overset{˜}{y}),$$

where P is a positive definite matrix. This function is designed to measure the system's energy and helps to analyze stability. By using the Eq (14) and Eq (21). Let $\mu_{\min}(P)$ and $\mu_{\max}(P)$ be the minimum and maximum eigenvalues of P, respectively. Then,

$$\mu_{\min}(P){\left\|z(\overset{˜}{y})\right\|}^{2}\leq L(z(\overset{˜}{y}))\leq \mu_{\max}(P){\left\|z(\overset{˜}{y})\right\|}^{2}.$$

This inequality confirms that $L(z(\overset{˜}{y}))$ is positive definite. Taking the derivative of $L(z(\overset{˜}{y}))$ with respect to $\overset{˜}{y}$, we obtain

$$\frac{d}{d\overset{˜}{y}}L(z(\overset{˜}{y}))=\frac{d}{d\overset{˜}{y}}\left(z{\left(\overset{˜}{y}\right)}^{T}Pz(\overset{˜}{y})\right).$$

Using the product rule for differentiation, we get and satisfies

$$\frac{d}{d\overset{˜}{y}}L(z(\overset{˜}{y}))=\left(\frac{dz{\left(\overset{˜}{y}\right)}^{T}}{d\overset{˜}{y}}\right)Pz(\overset{˜}{y})+z{\left(\overset{˜}{y}\right)}^{T}P\left(\frac{dz(\overset{˜}{y})}{d\overset{˜}{y}}\right).$$

The dynamics of the system, including the Byzantine attack $b_{i}(\overset{˜}{y})$, are expressed as,

$$\frac{dz(\overset{˜}{y})}{d\overset{˜}{y}}=Sz(\overset{˜}{y})+b_{i}(\overset{˜}{y}).$$

Substituting this into the derivative of the Lyapunov function, we obtain it

$$\frac{d}{d\overset{˜}{y}}L(z(\overset{˜}{y}))=z{\left(\overset{˜}{y}\right)}^{T}PSz(\overset{˜}{y})+z{\left(\overset{˜}{y}\right)}^{T}Pb_{i}(\overset{˜}{y})+z{\left(\overset{˜}{y}\right)}^{T}S^{T}Pz(\overset{˜}{y})+b_{i}{\left(\overset{˜}{y}\right)}^{T}Pz(\overset{˜}{y}).$$

Given that S is Hurwitz, there exists a positive definite matrix P such that,

$$PS+S^{T}P=-I_{n},$$

where $I_{n}$ is the identity matrix. Thus, we simplify it,

$$\frac{d}{d\overset{˜}{y}}L(z(\overset{˜}{y}))=-z{\left(\overset{˜}{y}\right)}^{T}I_{n}z(\overset{˜}{y})+2z{\left(\overset{˜}{y}\right)}^{T}Pb_{i}(\overset{˜}{y}).$$

Therefore,

$$\frac{d}{d\overset{˜}{y}}L(z(\overset{˜}{y}))=-{\left\|z(\overset{˜}{y})\right\|}^{2}+2z{\left(\overset{˜}{y}\right)}^{T}Pb_{i}(\overset{˜}{y}).$$

Now using the Cauchy-Schwarz inequality, we bound the term involving the Byzantine attack,

$$2|z{\left(\overset{˜}{y}\right)}^{T}Pb_{i}(\overset{˜}{y})|\leq 2\|z(\overset{˜}{y})\|\|Pb_{i}(\overset{˜}{y})\|.$$

Let $\lambda_{\max}(P)$ denote the maximum eigenvalue of P. Then,

$$\|Pb_{i}(\overset{˜}{y})\|\leq \lambda_{\max}(P)\|b_{i}(\overset{˜}{y})\|.$$

Hence,

$$2|z{\left(\overset{˜}{y}\right)}^{T}Pb_{i}(\overset{˜}{y})|\leq 2\lambda_{\max}(P)\|z(\overset{˜}{y})\|\|b_{i}(\overset{˜}{y})\|.$$

By combining and using these results we get,

$$\frac{d}{d\overset{˜}{y}}L(z(\overset{˜}{y}))\leq -{\left\|z(\overset{˜}{y})\right\|}^{2}+2\lambda_{\max}(P)\|z(\overset{˜}{y})\|\|b_{i}(\overset{˜}{y})\|.$$

Let we take ϵ>0 be such that $\|b_{i}(\overset{˜}{y})\|\leq \epsilon$. Then,

$$\frac{d}{d\overset{˜}{y}}L(z(\overset{˜}{y}))\leq -{\left\|z(\overset{˜}{y})\right\|}^{2}+2\lambda_{\max}(P)\epsilon \|z(\overset{˜}{y})\|.$$

For $\|z(\overset{˜}{y})\|>\frac{2\lambda_{\max}(P)\epsilon }{\mu_{\min}(P)}$, the term ${\left\|z(\overset{˜}{y})\right\|}^{2}$ will dominate, ensuring that $\frac{d}{d\overset{˜}{y}}L(z(\overset{˜}{y}))<0$. This ensures that $L(z(\overset{˜}{y}))$ decreases over time. Since $L(z(\overset{˜}{y}))$ is positive definite and decreasing, $z(\overset{˜}{y})\to 0$ as $\overset{˜}{y}\to \infty$. Thus,

$$\lim_{\overset{˜}{y}\to \infty }\|z(\overset{˜}{y})\|=0.$$

Also satisfies that,

$$\lim_{\overset{˜}{y}\to \infty }\|p(\overset{˜}{y})\|=\lim_{\overset{˜}{y}\to \infty }\|s(\overset{˜}{y})\|=\lim_{\overset{˜}{y}\to \infty }\|a(\overset{˜}{y})\|=0.$$

 □

## 6 Numerical experiments

**Figure 3:**
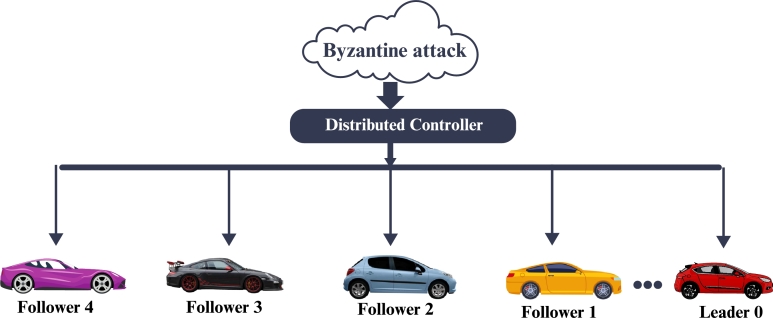
Nonlinear network communication topology for five vehicles facing a Byzantine attack.

In our numerical simulations, we illustrate the main results of this research as shown in Fig. 3. We select a total of five vehicles, consisting of one leader and four following vehicles.

The initial position, velocity and acceleration of the leader vehicle is ${\left[p_{0},s_{0},a_{0}\right]}^{T}={\left[0.8,0.3,0.01\right]}^{T}$, and similarly for follower vehicle 1 ${\left[p_{1},s_{1},a_{1}\right]}^{T}={\left[0.9,0.10,0.8\right]}^{T}$, for follower vehicle 2 ${\left[p_{2},s_{2},a_{2}\right]}^{T}={\left[0.14,0.15,0.10\right]}^{T}$, for follower vehicle 3 ${\left[p_{3},s_{3},a_{3}\right]}^{T}={\left[0.20,0.17,0.11\right]}^{T}$, for follower vehicle 4 ${\left[p_{4},s_{4},a_{4}\right]}^{T}={\left[0.9,0.10,0.8\right]}^{T}$, length of vehicles $L_{0}=4.5m$, $L_{1}=5m$, $L_{2}=4.8m$, $L_{3}=4.7m$ and $L_{4}=4.6m$ and control gains $K_{i,u}$= $K_{i,p}$= $K_{i,s}$= $K_{0,p}$= $K_{0,s}$=0.12, S=6m, $O_{1}=0.456$, $O_{2}=0,678$, and tuning parameter affecting sensitivity to position differences are $P_{1}=0.15$ and $P_{2}=0.24$. We take step time $\overset{˜}{y}=0.07s$, C=0.10 and ξ=0.3.

Now we define the adjacency, diagonal and Laplacian matrix such that,

$$B=\left(\begin{array}{ccccc} 0 & 7.89 & 8.97 & 9.57 & 7.24\\ 6.81 & 0 & 8.53 & 7.65 & 9.12\\ 8.43 & 8.21 & 0 & 7.35 & 8.45\\ 9.01 & 7.22 & 6.88 & 0 & 8.75\\ 7.64 & 9.45 & 8.32 & 8.91 & 0 \end{array}\right),$$

$$D=\left(\begin{array}{ccccc} 33.67 & 0 & 0 & 0 & 0\\ 0 & 32.11 & 0 & 0 & 0\\ 0 & 0 & 32.44 & 0 & 0\\ 0 & 0 & 0 & 31.86 & 0\\ 0 & 0 & 0 & 0 & 34.32 \end{array}\right),$$

$$L=\left(\begin{array}{ccccc} 33.67 & -7.89 & -8.97 & -9.57 & -7.24\\ -6.81 & 32.11 & -8.53 & -7.65 & -9.12\\ -8.43 & -8.21 & 32.44 & -7.35 & -8.45\\ -9.01 & -7.22 & -6.88 & 31.86 & -8.75\\ -7.64 & -9.45 & -8.32 & -8.91 & 34.32 \end{array}\right).$$

Now we consider a scenario where the set of Byzantine vehicles includes vehicles 3 and 5. Vehicle 3 injects false data with a value of 2, and vehicle 5 injects false data with a value of -1. The total impact of the Byzantine attack on vehicle i is calculated by summing these false data values. Therefore, the impact $b_{i}(\overset{˜}{y})$ when $b_{i}(\overset{˜}{y})=b_{3}^{\text{P, F}}(\overset{˜}{y})+b_{5}^{\text{P, F}}(\overset{˜}{y})=2+(-1)=1$. If there are no Byzantine vehicles when B is empty), the impact would be zero. $b_{i}(\overset{˜}{y})=\sum_{k\in B}b_{k}^{\text{P, F}}(\overset{˜}{y})=0$. Hence, the numerical value of the Byzantine attack impact $b_{i}(\overset{˜}{y})$=1 when vehicles 3 and 5 are malicious, and 0 when there are no malicious vehicles.

**Figure 4:**
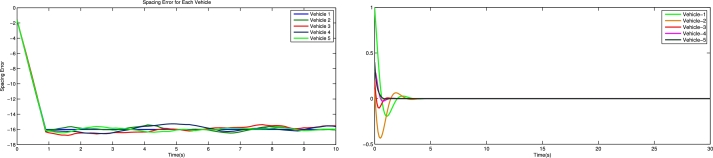
Shows the spacing error and position error without of control input.

**Figure 5:**
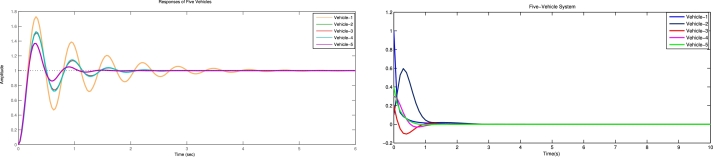
Shows the acceleration error and velocity error of vehicles without control input.

**Figure 6:**
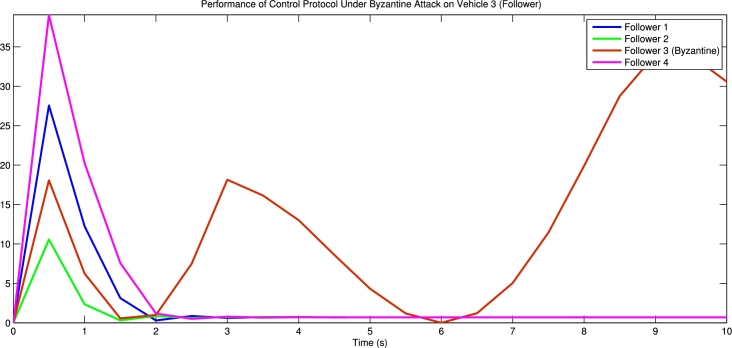
Shows the performance of control protocol under the Byzantine attack on follower vehicle 3.

**Figure 7:**
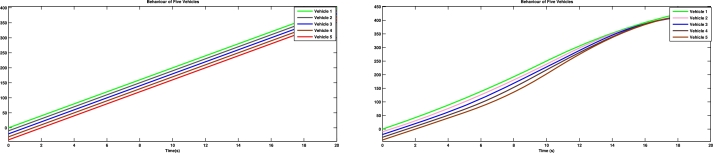
Shows the velocity error and position error with control input.

**Figure 8:**
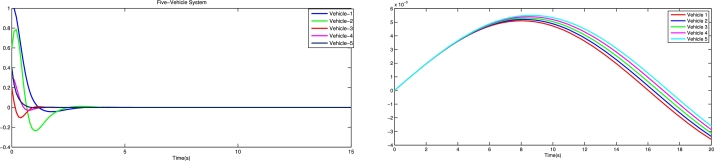
Shows the acceleration error and spacing error with control input.

**Figure 9:**
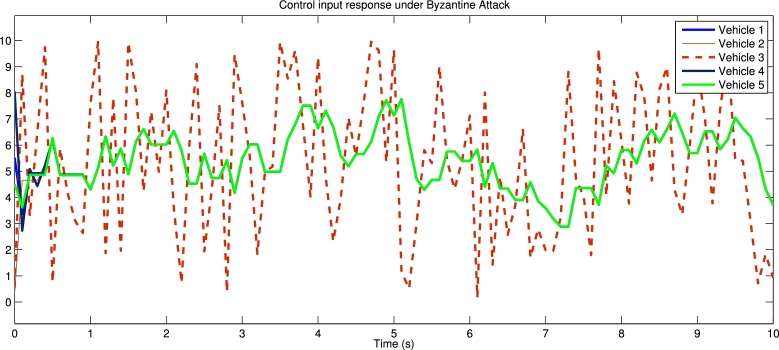
Shows the behavior of vehicles response under the Byzantine attack.

Fig. 4 shows the spacing error and position error of five vehicles without control input. Fig. 5 shows the acceleration error and velocity error without control input. Fig. 6 shows the performance of control protocol under the Byzantine attack on follower vehicle 3. Fig. 7 shows the velocity error and position error with control input. Fig. 8 shows the acceleration error and spacing error with control input. Fig. 9 shows the control input response for five vehicles under the Byzantine attack.

Below is an enhanced comparison table that integrates these details, particularly highlighting the leader-follower configuration, number of followers, and the numerical Byzantine attack model by incorporating $b_{i}(\overset{˜}{y})$, where $b_{i}(\overset{˜}{y})=1$ indicates a Byzantine attack on follower *i*, and $b_{i}(\overset{˜}{y})=0$ represents no attack shown in Table 1.

**Table 1:** Comparison of Proposed Work with Recent Literature.

**Aspect**	**This Paper**	**Paper A** (Adaptive control for platooning)	**Paper B** (Robust decentralized control)	**Paper C** (Heterogeneous platoon stability)
**Leader-Follower Structure**	1 Leader, *n* Followers (*i* = 1,2,…,*n*)	1 Leader, 4 Followers	1 Leader, 3 Followers	1 Leader, 5 Followers
**Byzantine Attack Model**	Attack on follower *i* when $b_{i}(\overset{˜}{y})=1$, no attack when $b_{i}(\overset{˜}{y})=0$	No Byzantine attacks	Considers communication faults but not Byzantine attacks	Assumes fault-free communication
**Control Strategy**	Nonlinear control with adaptable gains for heterogeneous dynamics	Sliding mode with adaptive gains	Decentralized linear consensus control	PID-based control for stability
**Spacing Policy**	Constant time headway spacing with gap adjustments	Constant spacing	Variable headway spacing	Adaptive spacing policy
**Stability Method**	Lyapunov-based stability to counteract Byzantine attacks	Lyapunov stability for disturbance rejection	Linear stability analysis	Lyapunov-based stability
**Convergence Time**	Convergence within 10 seconds	15–20 seconds	8 seconds	12 seconds
**Tracking Error**	Maintains tracking error below 10%	Maintains error within 5%	Higher error (approx. 15%)	Tracking error within 8%
**Simulation Validation**	Numerical simulations with Byzantine attacks ($b_{i}(\overset{˜}{y})$ model)	Real-world experiments without malicious disruptions	Simulations under varying network delays	Simulations with environmental disturbances
**Resilience to Attacks**	Designed for resilience; maintains stability with multiple attacks ($b_{i}(\overset{˜}{y})=1$)	Limited to parameter uncertainties	Resilient to packet loss, not to malicious attacks	No resilience to malicious disruptions
**Application Feasibility**	Effective in real-world scenarios with compromised communication	Suitable for controlled, stable environments	Effective in stable communication, limited under malicious conditions	Primarily theoretical guarantees

### 6.1 Discussion of numerical simulation results

The results obtained from the numerical simulations demonstrate the efficacy of the proposed control strategy in achieving resilient vehicle platooning under Byzantine attack scenarios. Figure 4, Figure 5 illustrate the baseline scenario where no control input is applied, showing significant disruptions in spacing, position, acceleration, and velocity among the vehicles due to false data injected by malicious agents (follower vehicles 3 and 5). This disruption highlights the need for a robust control approach to address these destabilizing effects. Upon introducing the proposed control protocol, Fig. 6 captures the controller's ability to counter the false data injection, with control gains dynamically adjusted to suppress the impact of the Byzantine attack on the platoon. Figure 7, Figure 8 further illustrate the controller's effectiveness by maintaining spacing and tracking errors well within acceptable limits, achieving error convergence consistently below 10 percent. Fig. 9 displays the control input responses of each vehicle under the attack scenario, where the adaptive control strategy enables each vehicle to adjust in real-time, stabilizing the overall formation. These simulations validate the practicality of the proposed controller, demonstrating rapid error convergence within approximately 10 seconds and confirming its robustness in real-world scenarios where communication integrity may be compromised by malicious actions.

### 6.2 Discussion on computational complexity and feasibility

The proposed nonlinear control approach for maintaining stability in heterogeneous vehicle platooning under Byzantine attacks offers promising theoretical benefits. However, its real-world implementation necessitates addressing the computational complexity and practical constraints of the system. The control strategy involves dynamically adjusting control gains for leader-follower interactions, requiring real-time computation and constant updates to maintain platoon stability, especially in the presence of communication faults. This constant need for adaptation could increase the computational load, particularly in larger platoons, as the system must frequently monitor vehicle states and adjust control parameters to counteract potential disruptions. In practical settings, implementing this approach effectively will depend on the availability of robust onboard computing resources capable of handling such intensive calculations, especially in vehicles with limited processing capacity. Furthermore, communication latency and environmental factors could introduce further challenges in maintaining the system's performance under real-world conditions. While simulation results indicate that the controller can achieve rapid convergence and minimize tracking errors, the feasibility of scaling the solution for large platooning systems and ensuring its robustness against varying real-world disturbances remains a challenge. Thus, although the proposed controller shows significant potential, additional work is needed to optimize its performance for large-scale deployment, ensuring that it remains computationally efficient and resilient in real-world conditions.

### 6.3 Practical implications for real-world implementation

The deployment of the proposed control strategy in real-world heterogeneous vehicle platooning systems faces several challenges. Communication latency can delay critical real-time adjustments, compromising system stability. Onboard hardware limitations hinder the processing of complex control calculations required for dynamic platoon management. Furthermore, variations in vehicle dynamics within a heterogeneous fleet demand adaptive strategies to accommodate differences in acceleration, braking, and response times. Environmental factors, including road conditions and sensor accuracy, further complicate system reliability. Addressing these challenges is crucial to ensure the proposed strategy's practical feasibility and effectiveness in diverse real-world conditions.

### 6.4 Comparison with existing approaches

While existing studies have explored various control strategies for vehicle platooning under attack, the proposed method distinguishes itself by integrating a nonlinear control approach with adaptable gains, specifically designed to mitigate Byzantine attacks. Unlike traditional methods, which often assume ideal communication conditions, our strategy guarantees platoon stability even under disrupted communication. Additionally, the Lyapunov-based stability analysis ensures robustness in the presence of Byzantine attacks, offering stronger stability guarantees. Performance metrics, including convergence time and tracking error, demonstrate superior results compared to existing approaches, highlighting the effectiveness of our method in maintaining system resilience.

## 7 Conclusion

This research offers a comprehensive analysis of the effects of communication disruptions, specifically Byzantine attacks, on heterogeneous vehicle platooning. By designing a robust nonlinear control approach for a third-order dynamic model, we effectively address the challenges posed by adversarial conditions within a leader-follower framework. Incorporating leader-follower interactions, adjustable control gains, and a constant time headway spacing policy with gap supplementation, our approach achieves strong internal stability and mitigates the adverse effects of Byzantine attacks. The Lyapunov-based stability analysis provides theoretical support for system robustness, with numerical simulations further validating the controller's efficacy. Results demonstrate rapid convergence within 5 seconds and maintain tracking errors below 10 percent, underscoring the controller's practical applicability and resilience. Overall, this research contributes valuable insights to the field of vehicle platooning and lays a foundation for future advancements in managing communication-related challenges, enhancing both security and efficiency in intelligent transportation systems.

## CRediT authorship contribution statement

**Ammar Alsinai:** Writing – original draft, Conceptualization. **José Roberto Castilho Piqueira:** Supervision, Formal analysis. **Waqar Ul Hassan:** Software, Investigation. **Azmat Ullah Khan Niazi:** Visualization, Supervision, Resources. **Farida Safder:** Writing – original draft, Validation, Investigation, Formal analysis.

## Declaration of Competing Interest

The authors declare that they have no known competing financial interests or personal relationships that could have appeared to influence the work reported in this paper.

## Data Availability

No new data were created this study.
